# Rapid multiple property determination from bulk materials libraries prepared from chemically synthesized powders

**DOI:** 10.1038/s41598-022-13691-3

**Published:** 2022-06-09

**Authors:** Li Ping Tan, V. Chaudhary, Z. Tsakadze, R. V. Ramanujan

**Affiliations:** School of Materials Science and Engineering, Nanyang Technological University, Singapore, 639798 Singapore

**Keywords:** Materials science, Materials chemistry

## Abstract

A variety of high-performance materials are utilized in electrical, electronic, and mechanical systems. Such systems account for a significant fraction of the world’s electricity consumption. The next generation of such systems urgently require new material compositions which possess a better combination of both structural and functional properties. Only accelerated methodologies can rapidly determine the required multiple property set. Hence, a range of iron–cobalt–nickel ternary alloy composition powders were chemically synthesized. Compositionally graded bulk materials libraries were prepared by spark plasma sintering of these powders. A multiple property set of the crystal structure, magnetic, mechanical, and electrical properties were determined for a range of compositions. This property set revealed that a good combination of magnetic and mechanical properties can be obtained from Fe_50_Co_40_Ni_10_, high electrical resistivity from Fe_54_Co_17_Ni_29_ and high saturation magnetization as well as high hardness from Fe_57_Co_29_Ni_14_. Thus, this multiple property library, developed by accelerated methodologies, can be utilized to identify new ternary compositions satisfying diverse property sets relevant to next generation systems.

## Introduction

Iron–cobalt–nickel based materials are widely used in many functional as well as structural components. For example, magnetic components are used in many applications, including rotating electrical machines, transformers, magnetic sensors and recording media^1–4^. These alloys can also possess excellent mechanical properties^5^. Hence, it is attractive to consider such ternary alloys for next generation devices and machines, as such systems will operate in harsh service conditions and require an adequate combination of mechanical, magnetic, and electrical properties. For example, next-generation high frequency, high torque, rotating electrical machines, such as motors, need materials with an attractive combination of functional and structural properties. Such electrical machines account for a significant fraction of the world’s electricity consumption. Hence, there is an urgent need to identify new material compositions with the appropriate property combination.

Some reported values of commercially available materials for such applications are: Permendur (Fe_49_Co_49_V_2_) based alloys have *M*_*s*_ ~ 228 emu/g, *H*_*c*_ ~ 0.4–1.25 Oe, *T*_*c*_ = 930 °C, electrical resistivity ~ 40 µΩ cm and Vickers hardness between 180 and 220 HV. Fe–Ni based alloys like Permalloy or Mumetall (Fe_15_Ni_80_Mo_5_ or Fe_14_Ni_77_Mo_4_Cu_5_) have *M*_*s*_ ~ 69 emu/g, *H*_*c*_ ~ 0.004–0.03 Oe, low *T*_*c*_ of 420 °C, electrical resistivity ~ 70 µΩ cm and Vickers hardness of 160 HV. Equimolar Fe–Ni (Fe_52_Ni_48_) have *M*_*s*_ ~ 157 emu/g, *H*_*c*_ ~ 0.05 Oe, low *T*_*c*_ of 450 °C, electrical resistivity ~ 48 µΩ cm and Vickers hardness of 120 HV^6^.

Literature data for the properties of Fe–Co–Ni ternary alloys are scattered and often confined to single property databases rather than property sets. The magnetic properties are available for equi-atomic alloys^7,8^ and a limited set of alloys of other compositions^1,9,10^, however, there are only a few reports on the crystal structure, mechanical and electrical properties as a function of ternary alloy composition^11–14^. Thus, there is no property library with integrated data on the magnetic, electrical and mechanical properties over a range of ternary Fe–Co–Ni alloy compositions. Traditional methods to develop such a property set will require unrealistically large time and resources. Hence, we deployed accelerated methodologies to create such a multiple property set.

A range of Fe–Co–Ni alloy compositions can be prepared for such accelerated studies by mechanical alloying^1,7–10^ but milling times of several hours are required to get suitable powder samples. Earlier^15^, the properties of a permalloy-cobalt system (Ni-21Fe)-xCo (x = 0, 20, 40 and 60) were studied by this methodology. Spark plasma sintering (SPS) was performed using ball-milled powders, and only Ni-rich and Co-rich regions of the Fe–Co–Ni system were investigated. On the other hand, wet chemical synthesis^16–19^, especially the hydrazine reduction method, is becoming increasingly popular as a facile and low-cost process to produce powders^20^. Hydrazine is a good metal reductant and produces nitrogen gas and water as by-products of the reaction, avoiding contamination issues which can degrade magnetic properties^21–23^.

We performed chemical reduction synthesis, via the hydrazine reduction, to produce powders of a range of Fe–Co–Ni alloy compositions. This was followed by the preparation of compositionally graded bulk samples (materials libraries) by spark plasma sintering (SPS) of these powders. A significant advantage of this accelerated development method is that a single bulk material library (ML) consists of adjacent layers of several distinct compositions. Hence, a single ML can be used for multiple property measurements for a range of compositions. For example, the X-ray diffraction (XRD) patterns of the various compositions present in a compositionally graded bulk ML were obtained in a single run using a suitable XRD equipment.

The structural, magnetic, mechanical and electrical property measurements of a variety of compositions were determined to develop a multi-property data set of Fe–Co–Ni alloys. Specific compositions with attractive combinations of mechanical, magnetic and electrical properties for these ternary alloys were identified. In this study, ternary compositions were distributed across the ternary space of the Fe–Co–Ni phase diagram to cover different regions—face-centered cubic (FCC), body-centered cubic (BCC) and two phase (FCC + BCC)—and alloy compositions with promising properties were identified.

## Experimental

### Fe–Co–Ni powder synthesis

Iron (II) chloride tetrahydrate (FeCl_2_·4H_2_O, 98%) and nickel (II) chloride hexahydrate (NiCl_2_·6H_2_O, 98%) from Alfa Aesar, cobalt (II) chloride hexahydrate (CoCl_2_·6H_2_O, 98%) and ethanol (EtOH, 99%) from Sigma Aldrich, hydrazine monohydrate (N_2_H_4_·H_2_O, 80% solution in water) from Merck, sodium hydroxide (NaOH) pellets from Schedelco and purified water (Type II^+^, Elga) were used as received. Sodium hydroxide pellets were dissolved in purified water to form a 4 M solution.

In a typical experiment for the synthesis of Fe–Co–Ni powder, the appropriate amounts of FeCl_2_·4H_2_O, CoCl_2_·6H_2_O and NiCl_2_·6H_2_O for a given ternary alloy composition were weighed, placed in a flask and stirred vigorously until the metal chlorides dissolved in the solvent (consisting of EtOH and purified water in the ratio 3:1). A 4 M NaOH solution was then added, followed by hydrazine monohydrate. The molar ratio of metal chlorides to NaOH to hydrazine monohydrate was approximately 1:2.5:16. The flask was then sealed, with a needle inserted to allow the evolved gases to vent, and the temperature was maintained at ~ 60 °C for 1 h. The black particles which formed were washed a few times with ethanol to remove the by-products. A permanent magnet was used to collect the black particles, which were then placed in a vacuum oven to form dry powders. The conversion yield to powders from each synthesis was more than 90%.

### Compositionally graded Fe–Co–Ni alloy using spark plasma sintering (SPS)

Compositionally graded bulk samples were prepared by SPS by consolidating the powders of a given composition as an individual layer, as described previously^15^. A tantalum foil was used as an inert spacer between each layer. Spark plasma sintering was performed in a Fuji Electronic Industrial SPS-211LX equipment at a vacuum level below 8 Pa under a pressure of 40 MPa at 950 °C for 15 min. Two vertically cut sections from the sample were prepared. One of the sections was labelled “as-SPS”, while the other section was annealed at 1000 °C for 2 h in 95% Ar + 5% H_2_ atmosphere and labelled “annealed”.

### Characterization techniques

The morphology of the chemically synthesized alloy powders was studied using a JEOL JSM-7600F field emission scanning electron microscope (FESEM). Elemental mapping was performed by an energy dispersive X-ray (EDX) spectrometer attached to the FESEM. The crystal structures of the as-SPS and annealed samples were determined by the XRD technique using a Bruker D8 Discover diffractometer (CuK_α_ radiation, λ = 0.154 nm). Phase fractions calculations were done via Rietveld refinement in TOPAS V6^24^ software. The Curie temperature (*T*_*c*_) was measured by a previously described method of conducting a thermogravimetric analysis (TGA) run of the sample while placing a permanent magnet near the TGA pan^15,25^, using a TA Instruments Q600 SDT. The magnetic properties were measured using a physical property measurement system (PPMS, EverCool-II, Quantum Design) equipped with a vibrating sample magnetometer (VSM) attachment.

The microhardness of the compositionally graded samples was measured using a Vickers hardness tester (Future-Tech) at a load of 1 kgf. A four-point probe tester (Keithlink) was used to obtain the resistivity (ρ), by applying the following equation^26,27^:

$$\rho = \frac{V}{I}.\frac{\pi t}{\mathrm{ln}(\frac{\mathrm{sinh}\left(\frac{t}{s}\right)}{\mathrm{sinh}\left(\frac{t}{2s}\right)})}$$

where V, I, *t* and s are the voltage, current, sample thickness and probe spacing respectively.

The graphical data presented in this work were plotted in Origin(Pro) 2020b^28^.

## Results and discussion

### Size and morphology of powders

**Figure 1:**
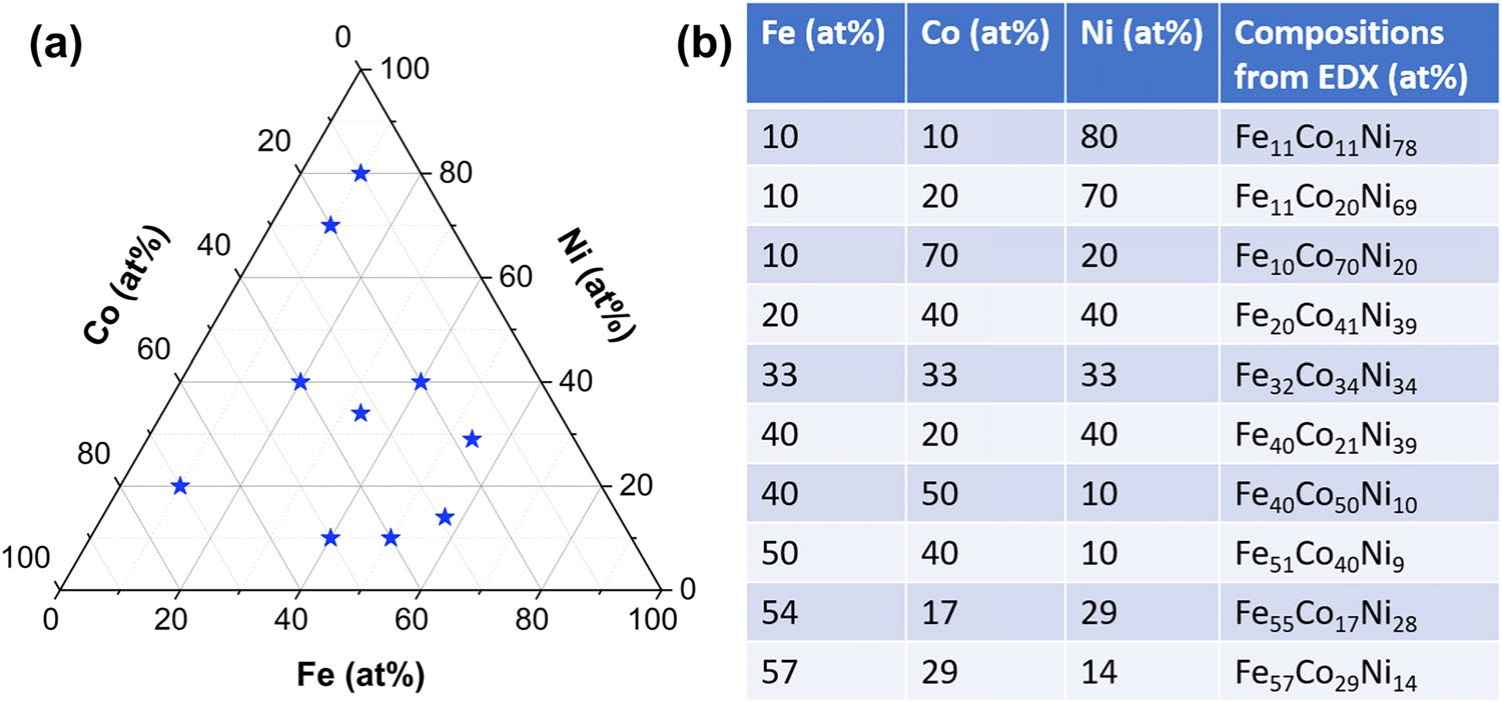
(**a**) Distribution of selected compositions shown on the ternary phase diagram, (**b**) Nominal and actual compositions of the Fe-Co–Ni ternary alloys.

Figure 1a shows the various compositions, in atomic percentage (at%), of Fe–Co–Ni powders synthesized in this work. Ten compositions from different regions of the ternary phase diagram were synthesized, and high throughput approaches were used to investigate the magnetic, mechanical and electrical properties. Sufficient mass of powders was produced from this synthesis method to form compositionally graded bulk samples. Figure 1b shows the nominal compositions as well as the composition values obtained using EDX. For ease of discussion, the samples will be referred to by their nominal compositions. The motivation to choose these specific compositions was to explore the various phase fields—face-centered cubic (FCC), body-centered cubic (BCC) and two phase (FCC + BCC) of the Fe–Co–Ni system. One of these compositions, Fe_54_Co_17_Ni_29_ is known as “Kovar” and is technologically important in the electronics industry^16,17^.

**Figure 2:**
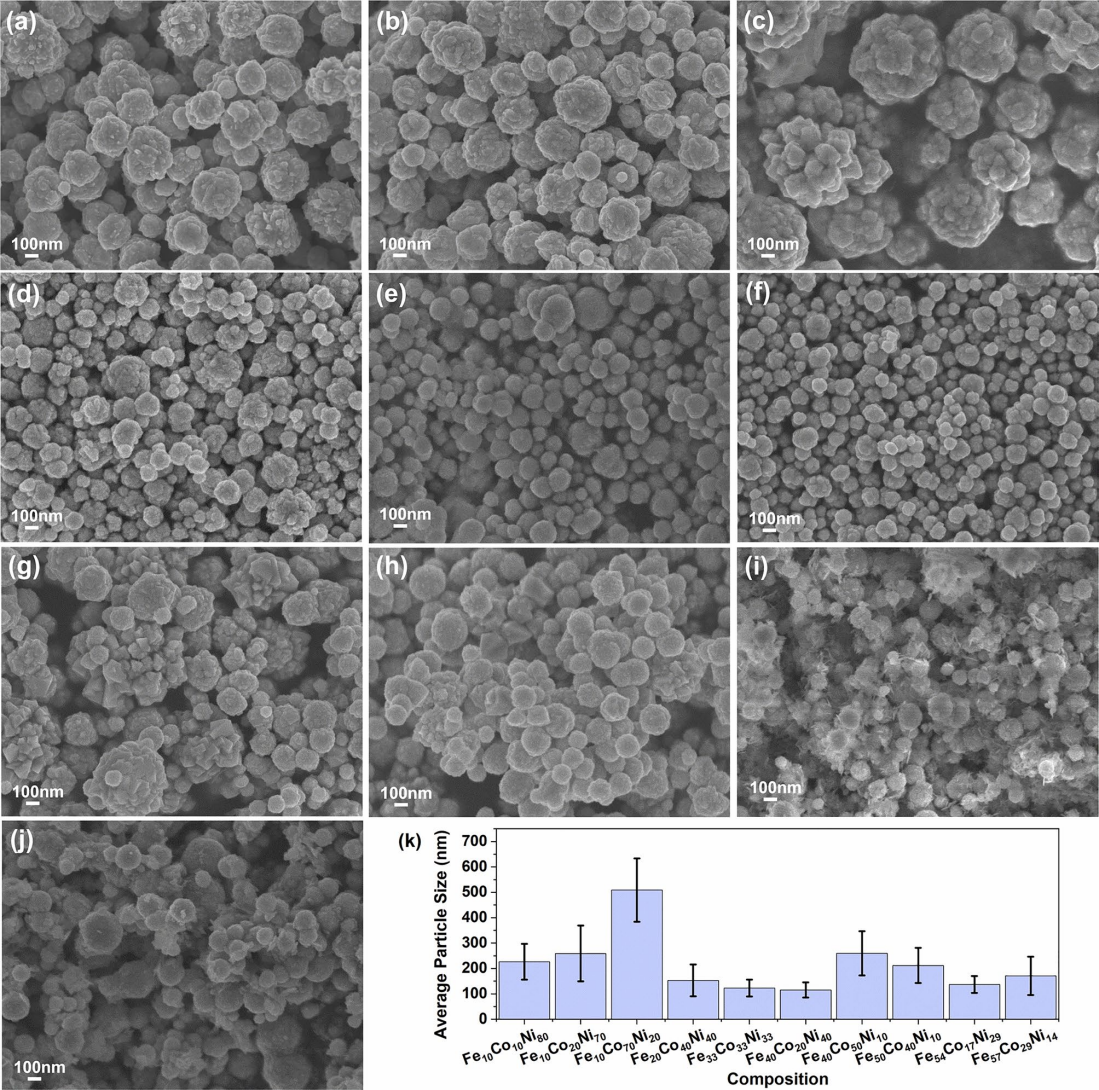
Size and morphology of the synthesized ternary alloy powders: (**a**) Fe_10_Co_10_Ni_80_, (**b**) Fe_10_Co_20_Ni_70_, (**c**) Fe_10_Co_70_Ni_20_, (**d**) Fe_20_Co_40_Ni_40_, (**e**) Fe_33_Co_33_Ni_33_, (**f**) Fe_40_Co_20_Ni_40_, (**g**) Fe_40_Co_50_Ni_10_, (**h**) Fe_50_Co_40_Ni_10_, (**i**) Fe_54_Co_17_Ni_29_, (**j**) Fe_57_Co_29_Ni_14_ and (**k**) average size of particles versus composition.

The scanning electron micrographs of the chemically synthesized ternary alloys are presented in Fig. 2a–j. The alloys generally formed spheres or spherulites and were in the particle size range of 55–750 nm. The average size of the particles with respect to composition is shown in Fig. 2k. Cobalt-rich compositions had larger particles sizes with more flower-like features—this morphological variation could be attributed to differences in the reduction rates of the chemical reduction reaction^18^.

### Structure and Phase Analysis of compositionally graded bulk materials libraries

**Figure 3:**
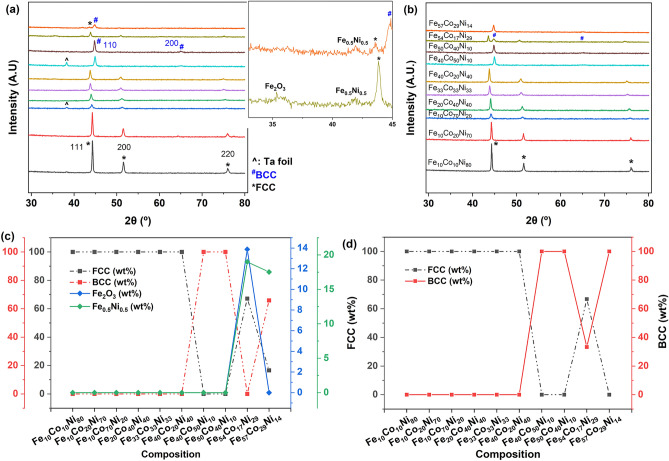
X-ray diffraction patterns of Fe–Co–Ni ternary alloys: (**a**) as-SPS, with the inset showing minority phases, (**b**) annealed at 1000 °C for 2 h in 95% Ar + 5% H_2_ atmosphere. Phase fractions in wt% of (**c**) as-SPS and (**d**) annealed Fe–Co–Ni ternary alloys, calculated via Rietveld refinement in TOPAS V6^24^.

Figure 3a,b shows the X-ray diffraction patterns of the as-SPS and annealed Fe–Co–Ni materials libraries. Depending on the composition, the crystal structures were either FCC, BCC or a mixture of both phases. In Fig. 3a, the as-SPS samples displayed varying degrees of crystallinity, with some compositions displaying diffraction peaks with much higher intensities. The diffraction peaks matched the 111, 200 and 220 peaks of the FCC phase, and the 110 and 200 peaks of the BCC phase. For the cases of Fe_54_Co_17_Ni_29_ and Fe_57_Co_29_Ni_14_, the intensities of the diffraction peaks were low, and minor phases of Fe_2_O_3_ and Fe_0.5_Ni_0.5_ were detected (see Fig. 3a inset).

After annealing (Fig. 3b), all samples exhibited sharp diffraction peaks, and the minor phases were absent. Annealing in gas containing hydrogen is beneficial for removing impurities such as oxygen^29^, and in dissolving the minor Fe_0.5_Ni_0.5_ phase back into the matrix. The crystal structures generally remained the same, but two compositions showed some changes: for Fe_54_Co_17_Ni_29_, an additional BCC phase formed, in addition to the initially present FCC phase, while for Fe_57_Co_29_Ni_14_, the FCC phase disappeared, leaving only the BCC phase. The weight % (wt%) of these phases in as-SPS and annealed samples are presented in Fig. 3c,d, respectively. As-SPS Fe_54_Co_17_Ni_29_ exhibited a FCC structure and contains ~ 14 wt% of Fe_2_O_3_ and 19 wt% of Fe_0.5_Ni_0.5_. As-SPS Fe_57_Co_29_Ni_14_ is two-phase, with FCC (16.6 wt%) and BCC (65.9 wt%) and contained 17.5 wt% Fe_0.5_Ni_0.5_. After annealing, 33.3 wt% of the BCC phase was present in Fe_54_Co_17_Ni_29,_ and the minor phases in both samples disappeared. This showed that heat treatment could be used to control the phases present in these ternary alloys. These compositions and phases match earlier reports for the FCC, BCC + FCC and BCC phase field regions^2,30^.

### Magnetic properties

**Figure 4:**
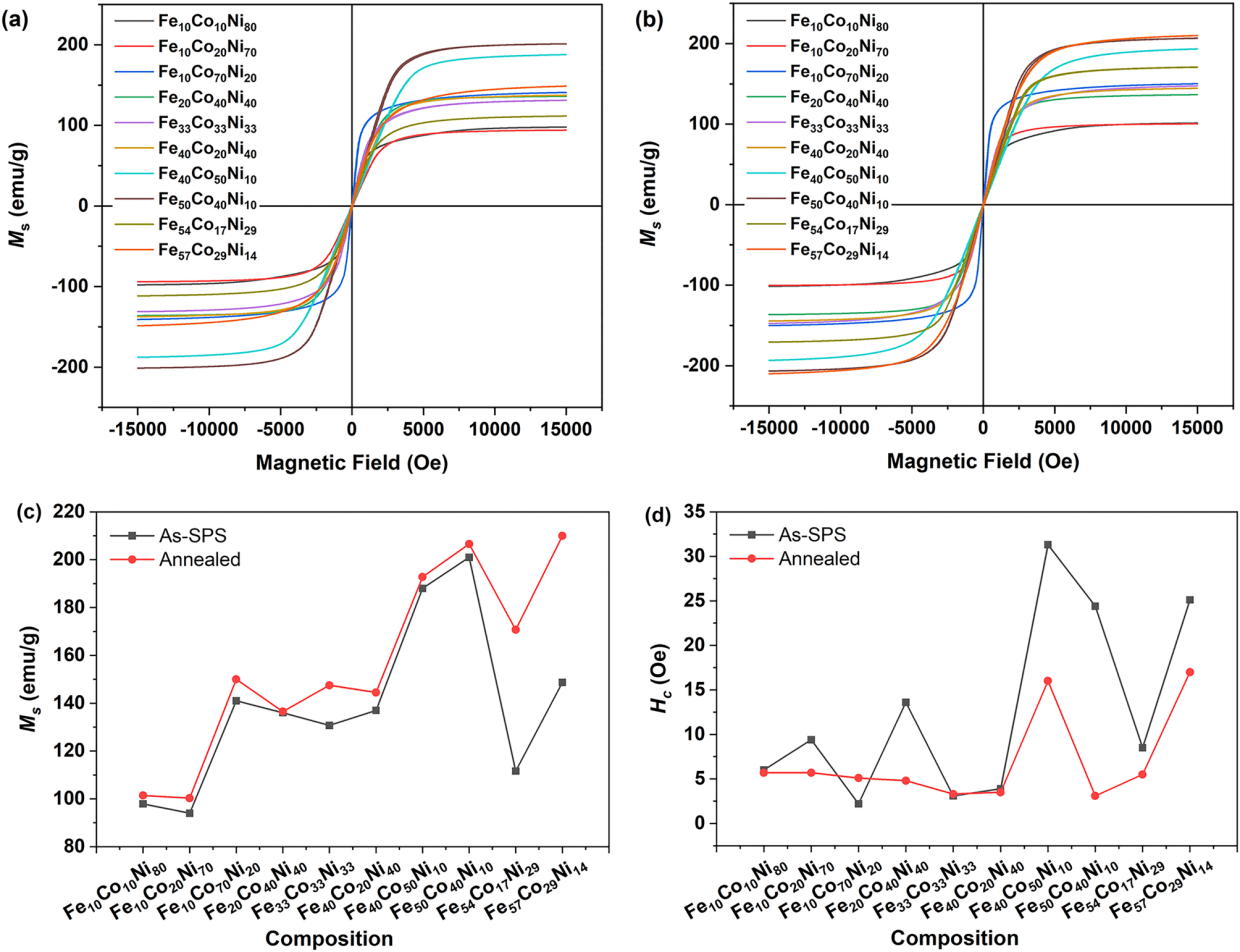
Field dependence of magnetization at room temperature for (**a**) as-SPS and (**b**) annealed samples, (**c**) variation of *M*_*s*_ with composition and (**d**) *H*_*c*_ as a function of composition for as-SPS and annealed ternary alloys.

Figure 4a,b shows the field dependence of magnetization at room temperature for the as-SPS and annealed samples. The variation of saturation magnetization (*M*_*s*_) with composition is shown in Fig. 4c. After annealing, the *M*_*s*_ increased; there was a 53% and 41% increase for the Fe_54_Co_17_Ni_29_ and Fe_57_Co_29_Ni_14_ compositions, respectively. This was due to the removal of the minority phases (of Fe_2_O_3_ and Fe_0.5_Ni_0.5_) in Fe_54_Co_17_Ni_29_ and Fe_57_Co_29_Ni_14_, as suggested by the XRD results.

For the alloy compositions with the same Fe content, those with higher Co content compared to Ni content exhibited higher *M*_*s*_ due to the larger atomic magnetic moment of Co compared to Ni (1.7 μB vs 0.6 μB)^31^. For the annealed samples, a high *M*_*s*_ of 207 emu/g and 210 emu/g was obtained for Fe_50_Co_40_Ni_10_ and Fe_57_Co_29_Ni_14_, respectively. The *M*_*s*_ values obtained in this work for equi-atomic Fe–Co–Ni were close to the reported value^18^ but higher than the reported values for Fe_40_Co_20_Ni_40_^16^ and Fe_50_Co_40_Ni_10_^17^, although it was noted that those samples were in powder form.

The coercivity (*H*_*c*_) was also obtained from the M–H curves, as shown in Fig. 4d. These values decreased or remained the same for most samples after annealing, except for Fe_10_Co_70_Ni_20_, in which case *H*_*c*_ increased after annealing. The most significant decrease in *H*_*c*_ after annealing was for Fe_50_Co_40_Ni_10_. At 3.1 Oe, it was also the lowest *H*_*c*_ value obtained. The samples in this work were in bulk form, hence the coercivity values of Fe_33_Co_33_Ni_33_, Fe_40_Co_20_Ni_40_ and Fe_50_Co_40_Ni_10_ were expected to be lower than those reported for the powder form^16–18^.

**Figure 5:**
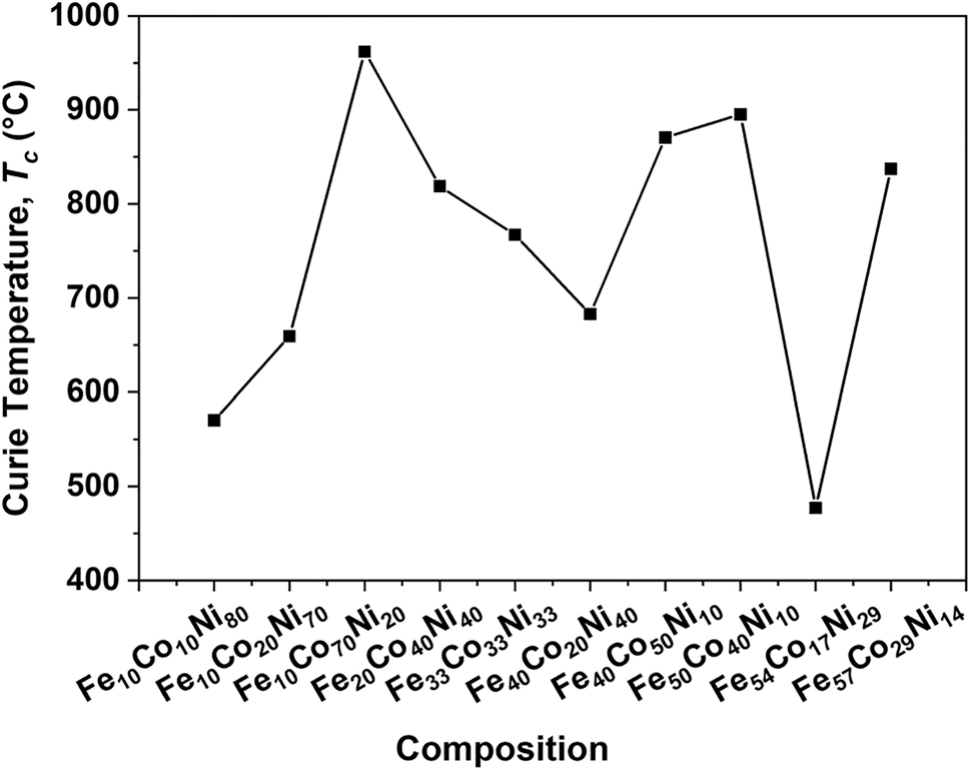
Curie temperature, *T*_*c*_, as a function of composition for annealed Fe–Co–Ni alloys.

Figure 5 shows the values of *T*_*c*_ versus composition in the ternary alloy samples. The *T*_*c*_ varied over a wide range, from 477 to 962 °C, making this study relevant to a variety of applications. Compositions that were rich in Fe and Co, with Ni between 10 and 20 at%, had higher *T*_*c*_ values. The *T*_*c*_ values for most samples matched closely with those reported in literature, while unique values were unavailable for Fe_40_Co_50_Ni_10,_ Fe_50_Co_40_Ni_10_ and Fe_57_Co_29_Ni_14_^32,33^. For these three compositions, more than one magnetic transition may occur, depending on the processing temperature, hence more than one value of *T*_*c*_ has been reported.

### Mechanical properties

**Figure 6:**
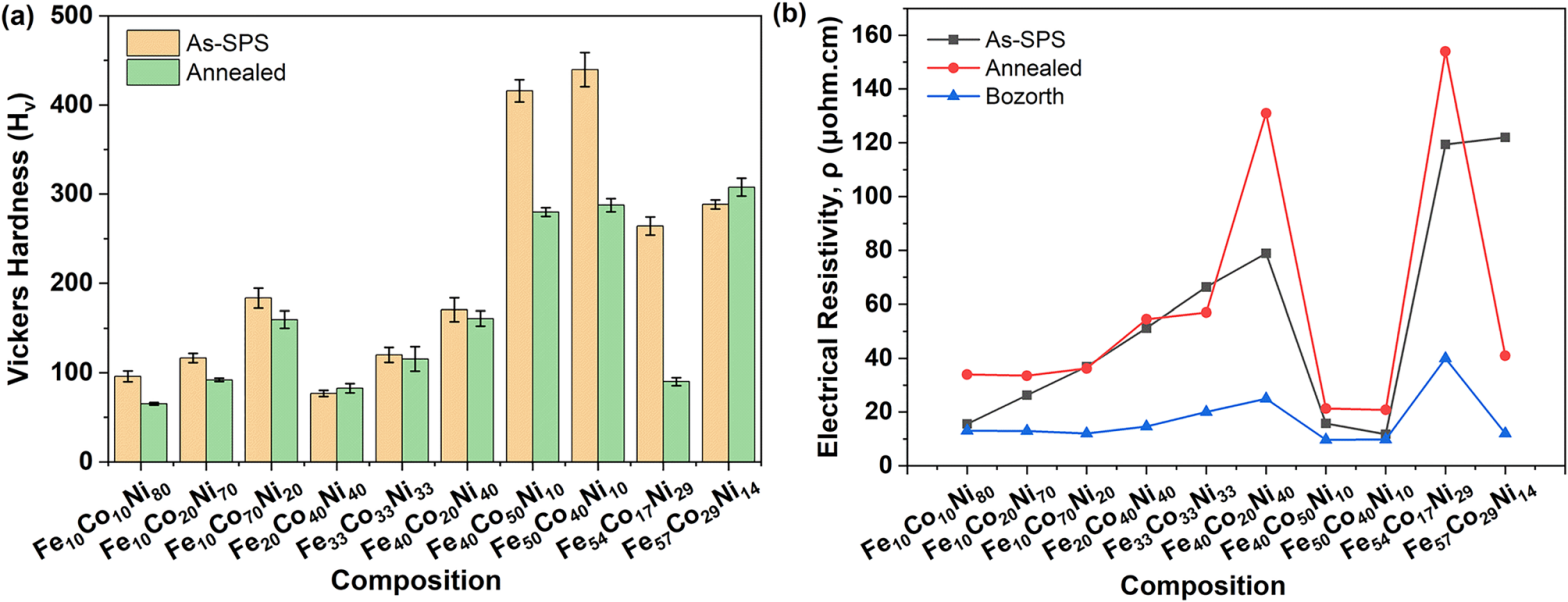
The (**a**) Vickers hardness, H_v_, and (**b**) electrical resistivity, ρ, of ternary alloys as a function of composition for as-SPS, annealed and reference values^33^.

Figure 6a,b shows the Vickers hardness (H_v_) and electrical resistivity (ρ) of the as-SPS and annealed samples. For the as-SPS samples, the microhardness varied greatly with composition, with the highest value of 439.5 H_v_ for Fe_50_Co_40_Ni_10_, followed closely by 415.9 H_v_ for Fe_40_Co_50_Ni_10_. After annealing, however, the microhardness decreased in all but two samples, Fe_20_Co_40_Ni_40_ and Fe_57_Co_29_Ni_14_, with these two compositions exhibiting a slight increase of 7.5% and 6.7%, respectively. For the case of Fe_57_Co_29_Ni_14_, this could be due to the disappearance of the FCC phase, which is known to be softer than the BCC phase^34^. A decrease in microhardness could also be due to lower residual stresses and defects^35^. This effect was most pronounced for Fe_54_Co_17_Ni_29_, where the magnitude of the decrease could be enhanced by the removal of the minority phases. The largest microhardness value (307.7 H_v_) after annealing occurred for the Fe_57_Co_29_Ni_14_ composition.

### Electrical properties

The electrical resistivity (ρ) of magnetic materials is important for controlling the power loss in rotating electrical machines. The eddy current loss is inversely proportional to the resistivity of the material. Figure 6b shows the values of electrical resistivity for the various compositions. Unexpectedly, the as-SPS samples generally exhibited values of electrical resistivity equal to or lower than those of the annealed samples, except for Fe_33_Co_33_Ni_33_ (where it was 16.5% higher) and Fe_57_Co_29_Ni_14._ Annealing led to the highest electrical resistivity value for the Fe_54_Co_17_Ni_29_ composition. Comparison to the reported literature values for Fe–Co–Ni alloys^33^ showed that the trend of electrical resistivity values closely matched the literature, but the absolute values were about 2–5 times higher, likely due to the different processing method used here for sample preparation as compared to the samples reported in the literature. It is also known that electrical resistivity can change as a function of annealing temperatures due to relaxation processes and phase transformations^36^; the high annealing temperature of 1000 °C may have led to higher resistivity values. Annealed Fe_57_Co_29_Ni_14_ exhibited the opposite trend for the change in microhardness and electrical resistivity compared with the other samples. This decrease in electrical resistivity could be attributed to a phase change, as shown by the XRD results.

**Figure 7:**
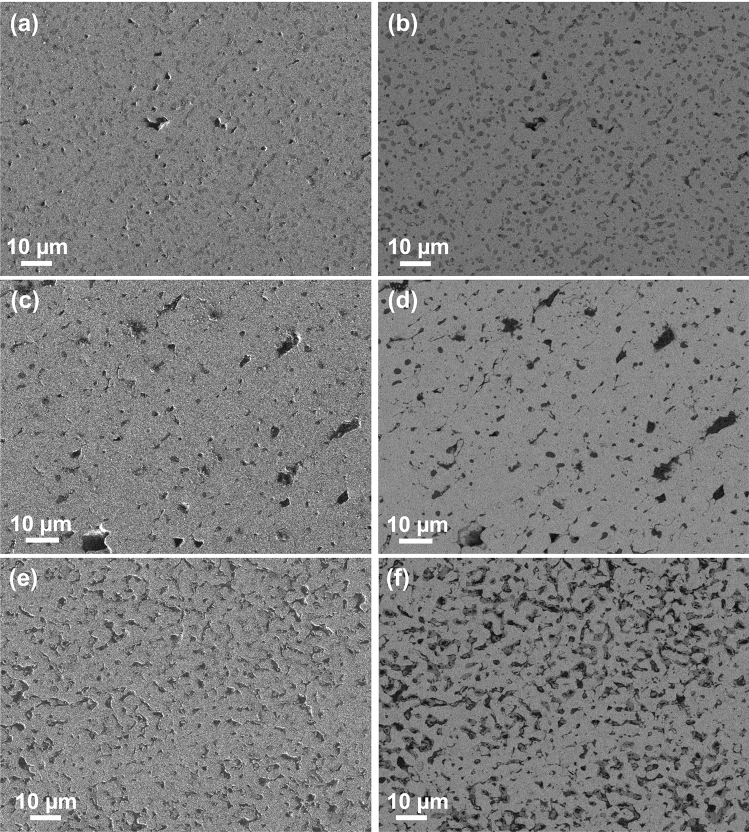
Secondary electron images and backscattered electron images of annealed (**a**, **b**) Fe_40_Co_20_Ni_40_, (**c**, **d**) Fe_54_Co_17_Ni_29_ and (**e**, **f**) Fe_57_Co_29_Ni_14_.

Scanning electron microscopy was performed on the three annealed samples which exhibited larger change in electrical resistivity—both secondary electron images and backscattered electron images were taken. For Fe_40_Co_20_Ni_40_ in Fig. 7a,b, pores of various sizes were observed. The increase in electrical resistivity was likely to be due to these pores. For Fe_54_Co_17_Ni_29_, the electrical resistivities of the minority phases was much higher for Fe_2_O_3_^37^ and slightly lower for Fe_0.5_Ni_0.5_ compared to the electrical resistivity of the ternary alloy^33^. Despite the removal of these minority phases, there was an increase in electrical resistivity due to the presence of multiple pores of various sizes and small and shallow cracks (Fig. 7c,d). For Fe_57_Co_29_Ni_14_, there was less porosity, but some surface roughness, as observed in Fig. 7e. Backscattered electron imaging in Fig. 7f showed that these pores were shallow and most of the alloy was still a continuous phase. Coupled with the removal of the Fe_0.5_Ni_0.5_ minority phase (which had higher electrical resistivity than this alloy composition^33^), the effect was a lower electrical resistivity value after annealing. The density of pores for Fe_40_Co_20_Ni_40_, Fe_54_Co_17_Ni_29_ and Fe_57_Co_29_Ni_14_ were measured using ImageJ^38^ and found to be ~ 1.1%, 4.2% and 4.6%, respectively. This porosity could be due to processing conditions and also the removal of impurity phases during annealing. Thus, changes in the phase fraction and defects, e.g. pores or cracks, can significantly affect measured electrical resistivity values of the samples. To reduce the porosity, higher sintering temperature and pressure can be used in SPS. The particle size of starting materials can also be tuned to minimize porosity in the sintered sample.

### Multiple property set obtained from the materials library

**Figure 8:**
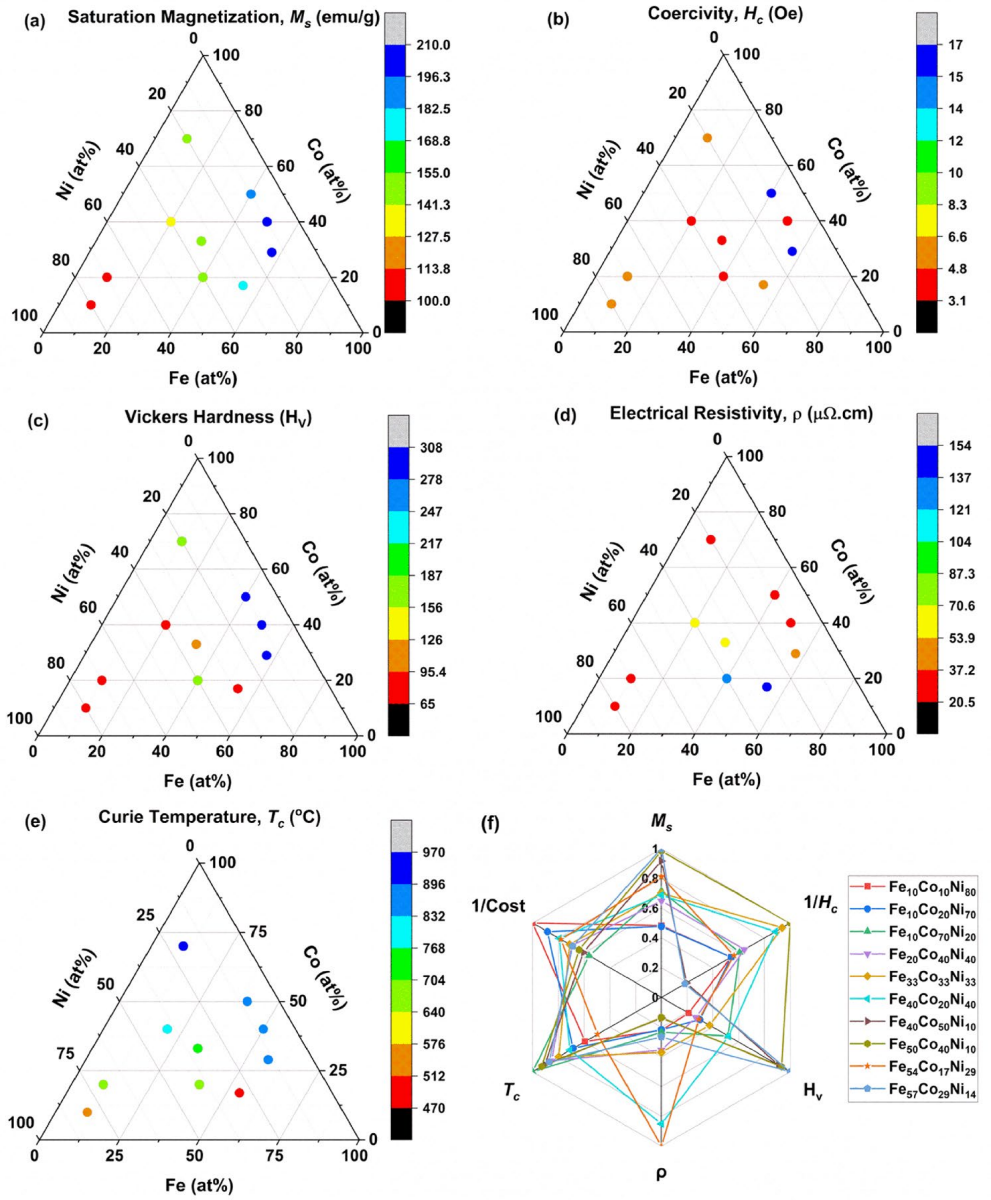
Colour maps of (**a**) saturation magnetization, *M*_*s*_, (**b**) coercivity, *H*_*c*_, (**c**) Vickers hardness, H_v_, (**d**) electrical resistivity, ρ, (**e**) Curie temperature, *T*_*c*_, for various compositions, and (**f**) radar plot for the materials properties and cost.

Figure 8a-e shows the colour maps comparing several properties after annealing, including *M*_*s*_, *H*_*c*_, H_v_, ρ, and *T*_*c*_. Fe_50_Co_40_Ni_10_ had attractive magnetic properties and microhardness values of 207 emu/g, 3.1 Oe and 287.5 H_v_, but its resistivity was low at 20.8 μΩ cm. Fe_54_Co_17_Ni_29_ had slightly lower values of *M*_*s*_ = 171 emu/g, *H*_*c*_ = 5.5 Oe and H_v_ = 90, but exhibited a desirable high resistivity of 154 μΩ.cm. Fe_57_Co_29_Ni_14_ had the highest *M*_*s*_ (at 210 emu/g) and H_v_ (at 307.7 HV) amongst the 3 compositions, but its *H*_*c*_ was somewhat higher at 17 Oe, and resistivity was relatively low at 41 μΩ cm.

This series of colour maps shows that accelerated methodologies can provide the multi-property set required for the identification of novel compositions possessing the required combination of properties. Broadly, the three compositions mentioned above exhibited promising combinations of properties.

Figure 8f shows the radar plot comparing normalized *M*_*s*_, *H*_*c*_, H_v_, ρ, *T*_c_ and cost values for the annealed samples. Of the three above-mentioned compositions, Fe_54_Co_17_Ni_29_ was the most cost effective. Selection can be readily performed for specific property requirements of a given application. For example, materials with higher resistivity could be considered for high frequency applications, while materials with higher *T*_*c*_ could be deployed in high temperature applications.

A high *M*_*s*_ of 207 emu/g was obtained for Fe_50_Co_40_Ni_10_, which is more than that of permalloy, the *T*_*c*_ was also 58% higher than permalloy^15^. The identification of such specific compositions in a vast ternary composition space underlines the advantage of our accelerated methodology. Further, chemical synthesis could be used to produce a wide variety of ternary alloy compositions.

In this accelerated methodology, we could quickly obtain valuable trends in the properties although a quantitative match with the conventional method was not always obtained. The absolute values of the mechanical and electrical properties of the compositions of interest were higher than previous literature on related permalloy-Co alloys^15^ due to differences in the compositions and the synthesis method. Electrical resistivity was a very sensitive function of several defects at various length scales and annealing had different effects on each composition. Hence there was a quantitative difference in the properties assessed by the accelerated and conventional methods.

### Validation

**Figure 9:**
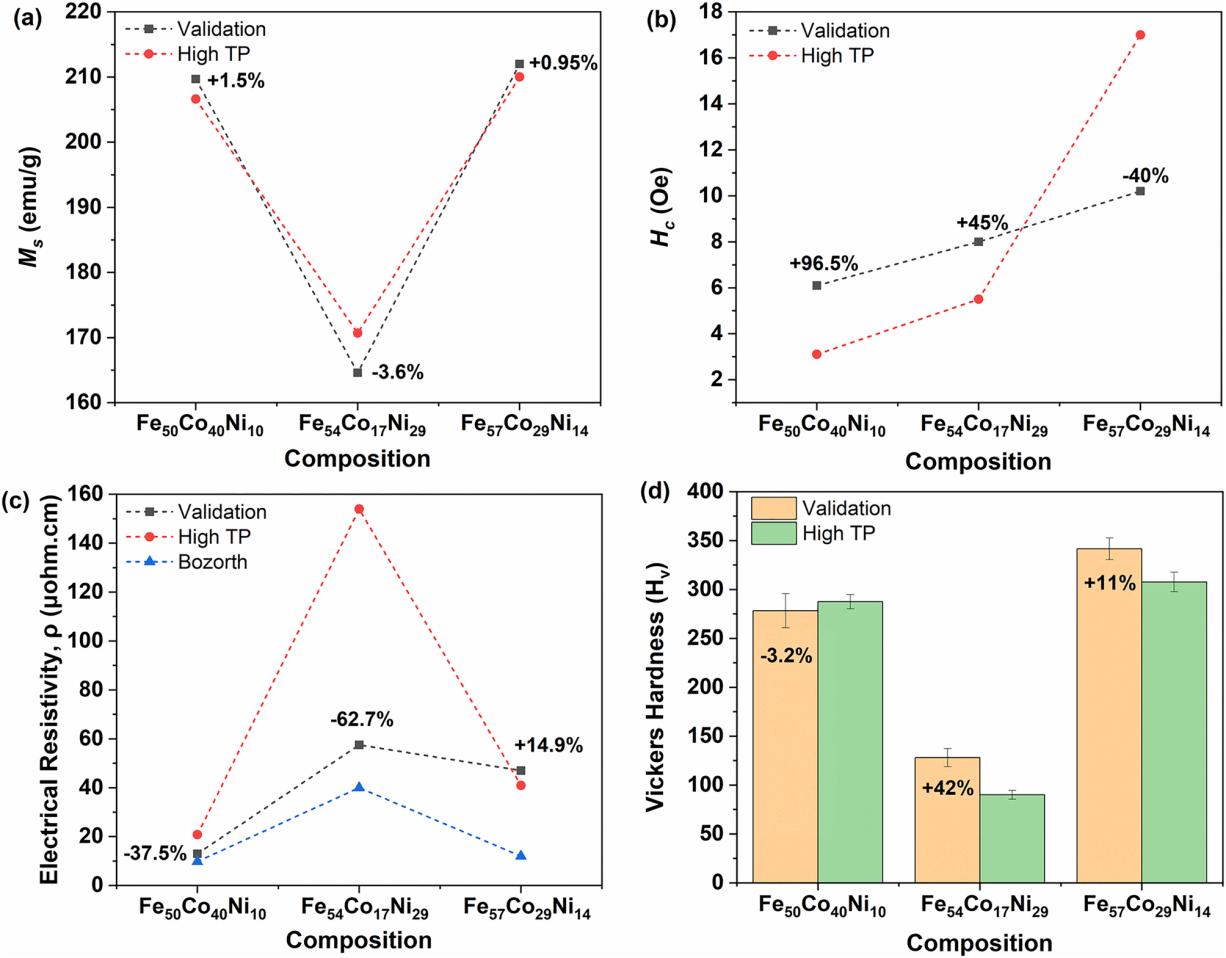
Validation results for (**a**) saturation magnetization, *M*_*s*_, (**b**) coercivity, *H*_*c*_, (**c**) electrical resistivity, ρ, and (**d**) Vickers hardness, H_v_.

Validation was performed on the three selected compositions from Fe_50_Co_40_Ni_10_ to Fe_57_Co_29_Ni_14_ that were predicted to exhibit a promising combination of properties. After synthesis and drying, the ternary alloy powders were compacted individually in SPS. The obtained pellets were then cut into half for annealing at 1000 °C. Earlier samples prepared by accelerated methodologies were labelled “High TP” for high throughput, validation samples were labelled “Validation”. As seen in Fig. 9a, the *M*_*s*_ of the samples closely matched for Fe_50_Co_40_Ni_10_ and Fe_57_Co_29_Ni_14_, while Fe_54_Co_17_Ni_29_ was 3.6% lower at 164.6 emu/g, likely due to small differences from the targeted composition of the sample. The coercivity values, *H*_*c*_, in Fig. 9b, varied across the three validation samples, compared to high throughput values: for Fe_50_Co_40_Ni_10_, it was 6.1 Oe, twice that of high throughput samples; for Fe_54_Co_17_Ni_29_ it was 45% higher at 8 Oe, and for Fe_57_Co_29_Ni_14_ it was 40% lower at 10.2 Oe. This highlighted the sensitivity of *H*_*c*_ to the details of the processing conditions.

In terms of the electrical resistivity (Fig. 9c), the same trend was maintained in the validation samples compared to the samples prepared via the high throughput method. However, the absolute values were lower here for Fe_50_Co_40_Ni_10_ and Fe_54_Co_17_Ni_29_ (at 37.5% and 62.7% respectively), but 14.9% higher for Fe_57_Co_29_Ni_14_. The unusually high electrical resistivity value obtained earlier for Fe_54_Co_17_Ni_29_ may have been due to pores or defects present in compositionally graded samples. This reinforced the earlier observation that electrical resistivity is sensitive to defects and pores of various length scales. These electrical resistivity values were higher than the values reported by Bozorth^33^.

Vickers hardness trends in Fig. 9d were similar to those obtained by the high throughput method. The value was 3.2% less than the high throughput value for Fe_50_Co_40_Ni_10_, 42% more than the high throughput value for Fe_54_Co_17_Ni_29_ and 11% more than the high throughput value for Fe_57_Co_29_Ni_14_. The high hardness of Fe_54_Co_17_Ni_29_ validation samples was likely to be due to less defects in the validation sample. Overall, the trends in the validation studies were like those seen in the accelerated methodology. Compared to the material properties of the commercially available materials, except for *H*_*c*_, which is higher, most of the other values obtained here are comparable.

## Conclusions

Hydrazine reduction synthesis was used to synthesize sufficient mass of powders for rapid multi-property evaluation of a range of ternary Fe–Co–Ni alloys. The powders were used to prepare compositionally graded bulk samples via SPS. These samples were evaluated by rapid structural characterization and multi-property assessment. A material property data set was developed, and a good balance of properties was identified in the composition region between Fe_50_Co_40_Ni_10_ and Fe_57_Co_29_Ni_14_. The crystal structures were either BCC or BCC + FCC. Compaction of ternary alloys into a bulk material and subsequent annealing in reducing gas improved magnetic properties. The Fe_50_Co_40_Ni_10_, Fe_54_Co_17_Ni_29_ and Fe_57_Co_29_Ni_14_ compositions were identified as possessing an interesting property mix. Results from the validation experiments were qualitatively like the high throughput results. Thus, an accelerated methodology to construct a multiple property data set from chemically synthesized powders processed into bulk samples (materials libraries) was successfully carried out and promising new alloy compositions identified.

## Data Availability

The data produced and analyzed during the current study are available from the corresponding author on reasonable request.
